# An Effective Single-Station Cooperative Node Localization Technique Using Multipath Spatiotemporal Information

**DOI:** 10.3390/s25030631

**Published:** 2025-01-22

**Authors:** Di Bai, Xinran Li, Lingyun Zhou, Chunyong Yang, Yongqiang Cui, Liyun Bai, Yunhao Chen

**Affiliations:** 1 College of Electronics and Information Engineering, South-Central Minzu University, Wuhan 430074, China; dibai@scuec.edu.cn (D.B.); xtxlxr20190678@163.com (X.L.); lingyunzhou@mail.scuec.edu.cn (L.Z.); cyyang@mail.scuec.edu.cn (C.Y.); cuiyq@mail.scuec.edu.cn (Y.C.); 2 Wuhan Shipboard Communication Institute, Wuhan 430079, China; berkeleyb@163.com; 3 Yunnan Key Laboratory of Unmanned Autonomous Systems, Kunming 650500, China; 4 School of Electrical and Information Engineering, Yunnan Minzu University, Kunming 650500, China

**Keywords:** collaborative sensor node, single-station localization, multipath spatiotemporal information, dual-antenna

## Abstract

Precise cooperative node localization is essential for the application of multifunctional integrated radio frequency (RF) sensor networks in military and civilian domains. Most geometric localization methods commonly rely on observation data from multiple receiving nodes or anchor points with known positions and synchronized clocks, producing complex system architectures and high construction costs. To address this, our paper proposes an effective single-station cooperative node localization technique, where the observation station only requires two antennas for operation. Leveraging prior knowledge of the geometry of surrounding structures, multiple virtual stations (VSs) are constructed by mining the spatiotemporal information contained in the multipath components (MPCs) to realize target positioning. The proposed method consists of two steps. In the first step, an unambiguous dual-antenna direction-finding algorithm is designed to extract the spatial information of MPCs and construct VSs, allowing a preliminary estimate of the source position (SP). In the second step, the path delays are extracted via matched filtering, while the spatiotemporal information is correlated based on the energy distribution for a more precise SP estimation. Simulations and experimental results demonstrate that our algorithm achieves high-precision single-station localization for a collaborative node, with positioning accuracy typically within 0.1 m.

## 1. Introduction

In recent years, multifunctional integrated RF sensor networks have been widely utilized in civil and military settings, including smart homes, fifth generation (5G) communications, environmental monitoring, battlefield deployment, and disaster monitoring [1,2,3,4,5]. In these scenarios, node location awareness is crucial for accurate monitoring, resource allocation, and task execution within sensor networks. The Global Navigation Satellite System (GNSS) can provide meter-level location information in open outdoor environments. However, in environments with poor propagation conditions, the GNSS often struggles to acquire high-quality navigation signals due to signal attenuation or obstruction [6,7,8]. With advancements in cooperative positioning technology (CPT), estimating the relative positions of nodes through information sharing among neighboring cooperative nodes or between nodes and anchor points with known locations has become a promising alternative, garnering widespread attention in both civilian and defense sectors, such as in navigation and surveillance systems [9,10,11,12].

Passive positioning, a technology attracting substantial attention in wireless positioning, can accurately locate a radiation source by analyzing and estimating the characteristics of its emitted electromagnetic waves [13]. According to the parameters commonly measured in collaborative positioning system, the positioning methods can be broadly categorized into the following types: received signal strength indicator (RSSI)-based positioning [14], angle of arrival (AOA)-based positioning [15], time of arrival (TOA)-based positioning [16], and time difference of arrival (TDOA)-based positioning [17]. The above measurement methods generally involve the use of multiple sensor-receiving nodes or nodes and anchor points to process data in order to achieve collaborative positioning. In contrast, the single-station positioning system offers several distinct advantages. Specifically, single-station positioning relies on a single observation platform to locate target radiation sources by capturing all positioning signals through the same antenna and analog-to-digital conversion chain, eliminating the need for synchronization protocols during the localization process, thus greatly simplifying the system. Additionally, single-station positioning does not depend on communication and data exchange between sensor platforms, making it particularly suitable for covert operations and highly mobile applications. Due to these commendable characteristics, single-station localization has been extensively studied as a promising technique for collaborative localization.

At present, scholars both domestically and internationally have conducted extensive research on the implementation and application of single-station positioning. The authors of Ref. [18] used a single receiver with an antenna array and several signal transponders at known locations to locate RF transmitters within a predefined search area. In Ref. [19], the authors implemented single-site positioning for a large-scale multiple-input multiple-output orthogonal frequency-division multiplexing (MIMO-OFDM) system based on fingerprint parameters of AOA and power delay profile (PDP), with numerical results demonstrating the method’s ideal performance. To provide reliable and accurate positioning in non-line-of-sight (NLOS) scenarios, the authors in Ref. [20] proposed a novel scheme that utilizes a single base station (BS) to simultaneously perform environmental sensing and NLOS user equipment (UE) positioning, leveraging the channel spatial sparsity and high spatial resolution of extremely large-scale antenna arrays provided by the near-field effect. For indoor applications, Ref. [21] investigated an AOA/TOA hybrid positioning system based on a single BS, capitalizing on the extensive bandwidth and subcarrier spacing of 5G signals to enhance positioning precision. Although the above studies have contributed to single-site positioning technology, the existing literature frequently relies on multi-antenna configurations to enhance positioning accuracy, consequently raising hardware costs and software measurement requirements. Additionally, in resource-constrained networks, such as multifunctional integrated RF sensor networks and other low-power wide-area Internet of Things (IoT) networks, equipping communication nodes with multiple dedicated antennas is impractical. A comparative analysis has been shown in Table 1.

Motivated by the above concerns, our work centers on minimizing antenna usage while still gathering enough information to achieve high-precision single-station positioning for collaborative nodes. Moreover, with the ongoing research into multipath utilization, the feasibility of constructing virtual nodes to extract target information and achieving positioning by classifying MPCs as friendly rather than hostile has been repeatedly validated [22,23,24]. Inspired by this, we propose a single-station passive positioning method based on multipath spatiotemporal information, which creates auxiliary virtual mirror observation points to extract real target information, allowing for position estimation of collaborative nodes using fewer antennas.

The main contributions are summarized as follows:

- We propose a novel and sophisticated single-station passive positioning algorithm for estimating the locations of cooperative nodes. This algorithm first constructs virtual mirror observation points based on MPCs for target detection. Then, by matching spatiotemporal information according to energy characteristics, it acquires the optimal solution for the positioning function, thereby achieving precise target location estimation.
- This paper introduces a dual-antenna unambiguous direction-finding technique to acquire the spatial information of MPCs, using channel ratios in a manner similar to circular synthetic aperture radar (SAR). This approach eliminates the need for clock synchronization, avoids the requirement for antenna array configurations, and imposes no specific waveform constraints, while still accurately estimating the directions of multiple incoming waves.
- Simulations and experimental results demonstrate that the positioning system presented in this paper achieves a positioning error within 0.1 m, while providing a more streamlined, cost-effective, and compact hardware implementation that requires only two receiving antennas in the sensor configuration to achieve high-precision location estimation of collaborative nodes. As a result, it sheds light on a more practical and versatile approach for real-world positioning applications.

The rest of this paper is structured as follows. Section 2 introduces the multipath-assisted positioning system that integrates spatiotemporal information adopted in this paper, and then constructs the corresponding detection algorithm based on this model. Section 3 verifies the algorithm’s effectiveness through simulations using MATLAB R2018b software and experiments in actual scenarios. Finally, the conclusions are presented in Section 4.

## 2. System Method

### 2.1. Mathematical Model

**Figure 1:**
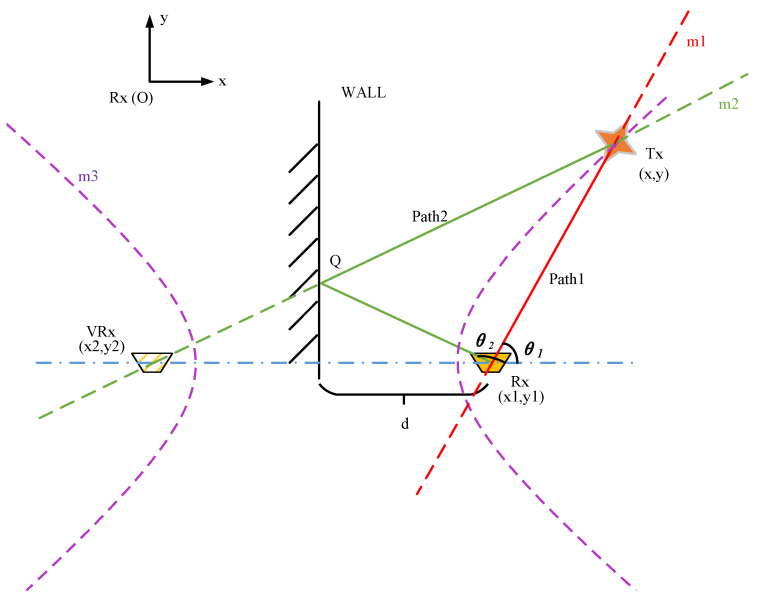
Positioning algorithm model diagram.

This paper establishes a model diagram, as shown in Figure 1, to illustrate the concept of the proposed positioning algorithm, where WALL represents a wall with known geographic information and Rx denotes the receiving device. As depicted in Figure 1, multipath effects arise during the transmission of electromagnetic waves from the cooperative node Tx, located at an unknown location, toward the receiving device Rx. The direct path, or the shortest path, is shown by the red polyline, Path1: Tx→Rx. Due to significant energy loss when the signal from Tx reaches the receiving end through paths with multiple reflections or wall diffraction, the impact of these paths on target position estimation is minimal. Therefore, this paper focuses on single-reflection multipath propagation scenarios. In Figure 1, a single-reflection path is present, where the electromagnetic wave reflects at point Q on the WALL and then propagates to the receiver, forming the propagation path indicated by the green polyline Path2: Tx→Q→Rx. Since Path2 corresponds to the specular reflection of the electromagnetic wave on WALL and has the same length as the path Tx→Q→VRx, the MPC along Tx→Q→Rx can be interpreted as the line-of-sight (LoS) signal from the transmitter Tx to the virtual receiver VRx. Based on the analysis of the multipath propagation process described above, the target point fulfilling both Path1 and Path2 is the intersection of the lines m1 and m2, which correspond to the paths Tx→Rx and Tx→Q→VRx, respectively. As a result, the target positioning problem is transformed into a problem of solving the straight-line equations of m1 and m2.

Since m1 and m2 pass through the known points Rx and VRx, respectively, the linear equations $M_{1}$ and $M_{2}$ for m1 and m2 can be determined by obtaining the corresponding angle parameters. To achieve this, a dual-antenna direction-finding algorithm is proposed in Section 2.3 to estimate the angle of the signal as it reaches the receiver after propagating along multiple paths. By applying this algorithm, a set of estimated arrival angles is obtained, including both the direct path angle $\theta_{1}$ and the first-order reflection angle $\theta_{2}$. With the known geometric information of buildings surrounding the receiver Rx as prior data, a Cartesian coordinate system centered at Rx can be established, as shown in Figure 1, where the coordinates of Rx, $\left(x_{1},\;y_{1}\right)$, are defined as $\left(0,\;0\right)$. The coordinates of VRx are given as $\left(x_{2},\;y_{2}\right)$, where $x_{2}=x_{1}-2d$ and $y_{2}=0$. The target Tx coordinates, $\left(x,\;y\right)$, can then be derived by solving the equations for m1 and m2, as shown in the following Equations:

(1) $$M_{1}=\frac{y-y_{1}}{x-x_{1}}-tan\theta_{1}=0,$$

(2) $$M_{2}=\frac{y-y_{2}}{x-x_{2}}+tan\theta_{2}=0.$$

Combine the vectors in Equations (1) and (2) as follows:

(3) $$M={\left[M_{1},M_{2}\right]}^{T}.$$

The analysis of the preceding equations reveals that the vector M depends on the target position coordinates $\left(x,\;y\right)$, as well as the AOAs for the direct path $\theta_{1}$ and the first reflection path $\theta_{2}$. Provided that the target position is accurately estimated and the AOA measurements for all paths are precise, the linear equations are satisfied, allowing us to deduce that:

(4) $${\left\|M\right\|}^{2}=0.$$

By solving Equations (1) and (2) simultaneously, we can obtain the coordinates x and y of the cooperative node, with $\theta_{1}$ and $\theta_{2}$ being known values derived from the direction-finding algorithm in Section 2.3.

### 2.2. Model Solution

In real-world experimental scenarios, the received data comprise not only the superimposed signals from the direct path and known reflections, but also those propagated through other scatterers. Furthermore, the algorithm presented in Section 2.3 is limited to obtaining the energy w and arrival angles φ from the received signal:

(5) $$\Theta =\left\{\left(w_{1},\varphi_{1}\right),\left(w_{2},\varphi_{2}\right),\cdots ,\left(w_{n},\varphi_{n}\right)\right\},w_{1}>w_{2}>\cdots >w_{n},$$

where n denotes the total number of paths. The direct path incurs minimal propagation loss and avoids destructive interference from multipath effects, thereby preserving a higher signal strength at the receiver. Thus, the angle $\varphi_{1}$ corresponding to the peak energy point can be considered as the estimated value ${\hat{\theta }}_{1}$ of the direct path’s arrival angle $\theta_{1}$. However, the value of $\theta_{2}$ cannot be determined directly. Therefore, when multiple reflective surfaces or scatterers surround the target, the critical challenge for the positioning algorithm proposed in this paper is to align the remaining AOA estimates in Θ with their corresponding VRxs, that is, determining the value of $\theta_{2}$. Taking Figure 1 as an example, the analysis will focus on Path2, where the signal is reflected from the known geographic feature WALL. We scan the arrival angles $\varphi_{r}\;\left(r\in \left[2,n\right]\right)$ from the set excluding the direct path and treat them as the estimated values ${\hat{\theta }}_{2}$ for $\theta_{2}$. The simultaneous Equations (1) and (2) can then be employed to determine a set of potential target coordinates as:

(6) $$Τ=\left\{\left({\hat{x}}_{i},{\hat{y}}_{i}\right)|{{\hat{\theta }}_{1}=\varphi_{1},\hat{\theta }}_{2}=\varphi_{i+1},i\in \left[1,n-1\right]\right\}.$$

Among the derived coordinates, only one corresponds to the actual value of $\theta_{2}$, representing the optimal solution for the target position. To identify this point, this paper proposes incorporating time-domain information as a constraint. Since the target is a cooperative object, the waveform information of the transmitted signal is available. Thus, this paper employs a matched filter based on cross-correlation to establish the relationship between the energy $w^{\prime }$ and the delay $\hat{\tau }$ for each path in the received signal as:

(7) $$\Gamma =\left\{\left(w_{1}^{\prime },{\hat{\tau }}_{1}\right),\left(w_{2}^{\prime },{\hat{\tau }}_{2}\right),\cdots ,\left(w_{n}^{\prime },{\hat{\tau }}_{n}\right)\right\},w_{1}^{\prime }>w_{2}^{\prime }>\cdots >w_{n}^{\prime }.$$

Notably, transmission and reception occur on separate devices without synchronized clocks, meaning that the delay obtained through the matched filtering method represents a relative delay. Since the direct path is the shortest and has the least energy loss, the time index of the peak energy point in the delay spectrum is taken as the relative delay estimate for the direct path. Obviously, within the same set of received data, the energy relationship of each path component of the signal remains constant. Therefore, the AOA estimation value φ can be mapped to the delay $\hat{\tau }$, yielding the set Κ as follows:

(8) $$Κ=\left\{\left(\varphi_{1},{\hat{\tau }}_{1}\right),\left(\varphi_{2},{\hat{\tau }}_{2}\right),\cdots ,\left(\varphi_{n},{\hat{\tau }}_{n}\right)\right\}.$$

The element $\left(\varphi_{1},{\hat{\tau }}_{1}\right)$ corresponding to the maximum energy in set Κ represents the AOA and relative delay estimate for the direct path. When re-observing the set Τ, for each potential target coordinate in Τ, there can be found a unique corresponding element in the set Κ. From this, we can further obtain:

(9) $${Κ}^{\prime }=\left\{\left({\hat{x}}_{1},{\hat{y}}_{1},{∆\hat{\tau }}_{1}\right),\left({\hat{x}}_{2},{\hat{y}}_{2},{∆\hat{\tau }}_{2}\right),\cdots ,\left({\hat{x}}_{n-1},{\hat{y}}_{n-1},{∆\hat{\tau }}_{n-1}\right)\right\},$$

where ${∆\hat{\tau }}_{m}={\hat{\tau }}_{m+1}-{\hat{\tau }}_{1}$ represents the delay difference between the $m+1\;\left(m\in \left[1,n-1\right]\right)$ path and the direct path. For Path1 and Path2, $d_{Tx,Q,Rx}-d_{Tx,Rx}=d_{Tx,Q,VRx}-d_{Tx,Rx}=C\;$(constant), where $d_{l}$ represents the length of the Path. The trajectory equation that satisfies the target $\left(x,\;y\right)$ can be regarded as a hyperbola $M_{3}$, with Rx and VRx as the focal points and the difference between Path1 and Path2 as the length of the semi-real axis. The detailed equation can be shown by:

(10) $$M_{3}(x,y,∆\tau )=\frac{{\left(x-\frac{x_{1}+x_{2}}{2}\right)}^{2}}{a^{2}}-\frac{{\left(y-\frac{y_{1}+y_{2}}{2}\right)}^{2}}{d^{2}-a^{2}}-1,$$

where $a=\frac{\left|d_{Tx,Q,Rx}-d_{Tx,Rx}\right|}{2}=\frac{\left|c\cdot ∆\tau \right|}{2}$, c represents the speed of light, and ∆τ represents the path delay difference between Path1 and Path2.

As both transmitting and receiving devices operate with independent, unsynchronized clocks, clock instability arises on both ends. Clock drift, which is mainly caused by factors such as temperature changes, voltage fluctuations, aging effects, and manufacturing process differences, is the main part of the instability. The magnitude of clock drift is predominantly influenced by the choice of reference source. In accordance with the IEEE 802.15.4a [25] standard, clock drift in general communication devices is permitted to reach a maximum of ±20 ppm. Based on this, the clock drift is modeled as follows:

(11) $${\hat{\tau }}_{1}=({1+e_{t})(1+e_{r})\tau }_{1},$$

(12) $${\hat{\tau }}_{m+1}=({1+e_{t})(1+e_{r})\tau }_{m+1},$$

where X and $\hat{X}$ represent the ideal and actual values, respectively, while t and r signify the transmitting and receiving devices, respectively. $e_{x}$ denotes the deviation from the standard frequency, typically expressed in ppm. Given that the drift magnitude is minimal, the second-order term $e_{t}e_{r}$ can be neglected. Consequently, the estimated time delay difference ∆τ can be expressed as:

(13) $${∆\hat{\tau }}_{m}={\hat{\tau }}_{m+1}-{\hat{\tau }}_{1}=\left(\tau_{m+1}-\tau_{1}\right)\left(1+e_{t}+e_{r}\right)={∆\hat{\tau }}_{m}+{∆\hat{\tau }}_{m}\cdot \left(e_{t}+e_{r}\right).$$

Typically, the path delay differences at the receiver are in the order of nanoseconds. Assuming ∆τ is 100 ns, and based on the error specifications outlined in IEEE 802.15.4a, the maximum clock drift $e_{t}+e_{r}$ can reach ±40 ppm. As a result, the corresponding error is computed as follows: 100 ns×±40 ppm=0.004 ns. Given that electromagnetic waves travel at the speed of light, the error introduced by asynchronous clock asynchrony is approximately 0.12 cm, which is negligible and meets the accuracy requirements for most indoor and outdoor positioning applications. Consequently, the influence of clock drift can be safely neglected in our proposed scheme.

Under the conditions of fixing the geographical location of the reflection surfaces, receiving devices, and virtual receiver, only one optimal solution exists that satisfies the above trajectory equations. At the same time, due to the limitations in measurement precision and resolution during actual measurements, the straight lines and hyperbola in the model cannot strictly intersect at a certain point, but instead converge within a constrained region. Based on the above description, the target position coordinates are:

(14) $$\left(\hat{x},\hat{y}\right)=\mathrm{arg min}\left(M_{3}({\hat{x}}_{i},{\hat{y}}_{i},{∆\hat{\tau }}_{i})\right),({\hat{x}}_{i},{\hat{y}}_{i},{∆\hat{\tau }}_{i})\in {Κ}^{\prime }.$$

To evaluate the performance of the positioning algorithm proposed in this paper, we use the root mean square error (RMSE) as a metric to quantify the geometric deviation between the actual and estimated positions [26]. The RMSE is calculated using the following formula:

(15) $$RMSE=\sqrt{\frac{1}{p}\sum_{j=1}^{p}\left[{\left(x-{\hat{x}}_{j}\right)}^{2}+{\left(y-{\hat{y}}_{j}\right)}^{2}\right]},$$

where p represents the number of measurements, $\left(x,\;y\right)$ denotes the actual coordinates of the target point, and $\left({\hat{x}}_{j},\;{\hat{y}}_{j}\right)$ represents the target position of the j*-*th measurement.

The above provides an overview of the single-station passive positioning algorithm driven by multipath spatiotemporal information fusion, where the direction-finding accuracy directly affects the final positioning accuracy and system performance. Traditional passive direction-finding systems, such as interferometer direction-finding [27,28,29], spatial spectrum direction-finding [30,31,32], and MIMO radar-based direction-finding techniques [33,34], typically require at least three antennas to form an array to receive signals in order to achieve a high angular resolution. Additionally, phase ambiguity should also be considered, which complicates the system design and increases costs. To better address the needs of collaborative node position estimation, a dual-antenna unambiguous direction-finding scheme is designed in this paper to extract the spatial information from multipath signals. The implementation principle of the algorithm will be detailed in the next section.

### 2.3. Extraction of Spatial Information Based on MPCs

**Figure 2:**
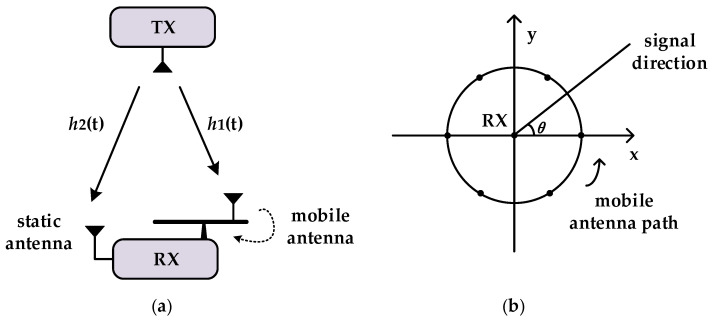
(**a**) Dual-antenna direction-finding system model; (**b**) circular synthetic aperture.

Ref. [35] introduces in detail the theory of how to construct multipath profiles using SAR. The core concept is to simulate a virtual array of multiple antennas by moving a single antenna. Based on this, this paper develops a SAR-like dual-antenna direction-finding system model diagram, as shown in Figure 2a, where transmission and reception are completed on independent devices. The receiving device consists of a stationary antenna and a mobile antenna, with the mobile antenna moving along a circle with a radius of r, similar to a circular SAR. As shown in the circular SAR model diagram of Figure 2b, assuming that the receiver at the origin aims to measure the power of the signal $P\left(\theta \right)$ received from the independent transmitter in the direction of the azimuth angle θ, according to the SAR formula, the value of $P\left(\theta \right)$ is:

(16) $$P\left(\theta \right)=\sum_{t}{\left|a_{f}\left(t,\theta \right)h\left(t\right)\right|}^{2},\;a_{f}\left(t,\theta \right)=e^{-j\frac{2\pi f}{c}rcos\left(\theta -\theta_{0}\left(t\right)\right)},$$

where $h\left(t\right)$ is the wireless channel from the transmitter to the mobile antenna at time t. Assuming that the frequency difference between the transmitter and the receiver is 0, $a_{f}\left(t,\theta \right)$ is the weight of the wireless channel of the mobile antenna at the azimuth angle θ at time t. By adjusting this weight, the signal strength in the incoming wave direction can be enhanced to achieve spatial filtering. In this paper, a circular SAR is employed, where the antenna rotates with a radius r. The specific value of $a_{f}\left(t,\theta \right)$ is given by $e^{-j\frac{2\pi f}{c}rcos\left(\theta -\theta_{0}\left(t\right)\right)}$, where $\theta_{0}\left(t\right)$ represents the angular position of the mobile antenna at time t.

In traditional SAR systems, where the same reference clock is used for both transmission and reception, the measured wireless channel value $\tilde{h}\left(t\right)$ of the mobile antenna is independent of the frequency. Therefore, it can be stated that $\tilde{h}\left(t\right)=h\left(t\right)$. However, in practical scenarios, where SAR is performed between independent transmitters and receivers, the measured wireless channel $\tilde{h}\left(t\right)$ will have $\tilde{h}\left(t\right)=h\left(t\right)e^{j\varphi \left(t\right)}$ due to position changes, carrier and sampling frequency offsets between the transmitters and receivers, and phase noise. To eliminate this accumulated phase $e^{j\varphi \left(t\right)}$, as shown in Figure 2a, we use both a stationary antenna and a mobile antenna for reception. Since the mobile antenna and the static antenna receive signals at the same time in the experiment, it can be considered that the two antennas are connected to the same reference clock. Moreover, given that the accumulated phase reflects the global characteristics of the system clock, rather than the deviation of the sampling time of the two antennas, the term $e^{j\varphi \left(t\right)}$ remains consistent. Consequently, the experiment is set up as follows:

(17) $${\tilde{h}}_{1}\left(t\right)=h_{1}\left(t\right)e^{j\varphi \left(t\right)},{\tilde{h}}_{2}\left(t\right)=h_{2}\left(t\right)e^{j\varphi \left(t\right)},$$

where $h_{2}\left(t\right)\approx h_{2}$ remains relatively constant over a short period. Additionally, in practical applications, the relative timing differences between the stationary and mobile antennas are usually small and stable over short periods, typically within the nanosecond range, having minimal impact on the final localization accuracy. As a result, there is no necessity to maintain additional relative timing between the stationary and mobile antennas. Thus, the wireless channel ratio $\frac{{\tilde{h}}_{1}\left(t\right)}{{\tilde{h}}_{2}\left(t\right)}=\frac{1}{h_{2}}h_{1}\left(t\right)$ acts as a constant multiple of the mobile channel, unaffected by phase accumulation from frequency offset and noise. Hence, $\frac{{\tilde{h}}_{1}\left(t\right)}{{\tilde{h}}_{2}\left(t\right)}=\frac{1}{h_{2}}h_{1}\left(t\right)$ can replace $h\left(t\right)$ in Equation (16) for calculation, effectively allowing SAR analysis of the incoming signal in the receiver without frequency offset and noise accumulation. Thus, we traverse θ, where $\theta \in \left[0,\pi \right]$, and calculate the corresponding $P\left(\theta \right)$. In the resulting graph, the angle corresponding to the beam with the largest amplitude is the incoming wave direction. It is worth mentioning that this method can identify multiple incoming wave directions. Specifically, in the spatial spectrum after data processing, a corresponding peak will appear at each incoming wave direction.

Algorithm 1 illustrates the steps of this SAR-like direction-finding algorithm.
**Algorithm 1:** Extraction of Spatial Information Based on MPCs
**Input:** Q data files, each containing K sample points of I/Q data from both moving and stationary antennas at q-th (1≤q≤Q) different angular position.
**Output:** A .mat file with spatial angles Degrees and corresponding power values Power.1.**for** each data file q=1 to Q **do**2. Read the file to extract the angular position $\theta_{0}(k)$ and 2K corresponding I/Q samples from both the moving antenna reception ${\tilde{h}}_{1}\left(k\right)$ and the stationary antenna reception ${\tilde{h}}_{2}\left(k\right)$.3. Store $\theta_{0}(k)$ as the key and ${\tilde{h}}_{1}\left(k\right)$, ${\tilde{h}}_{2}\left(k\right)$ as the value in the mapping table d{key}.4.**end**5.**for** each key in d **do**6. Extract the data ${\tilde{h}}_{1}\left(k\right)$ and ${\tilde{h}}_{2}\left(k\right)$ for K sample points, respectively.7. Compute the ratio $h_{r}\left(k\right)$ of ${\tilde{h}}_{1}\left(k\right)$ to ${\tilde{h}}_{2}\left(k\right)$ and store the result in the updated mapping table $d_{updte}\left\{key\right\}$, with the calculation of $h_{r}\left(k\right)$ performed as follows: $h_{r}\left(k\right)=\frac{1}{K}\sum_{k=1}^{K}\frac{{\tilde{h}}_{1}\left(k\right)}{{\tilde{h}}_{2}\left(k\right)}$.8.**end**9.**for** cos θ=−1 to 1, with a step size of 0.01 **do**10. **for** each key in $d_{updte}$ **do**11. Calculate the received power P(θ) at spatial angle θ based on Equation (16).12. **end**13. Store the current angle θ in the array Degrees, and the corresponding power P(θ) in the array Power.14.**end**15.Save the arrays Degrees and Power as a .mat file for further analysis.

After determining the arrival angle of the multipath signal by observing the peak of the spatial spectrum, this paper performs matched filtering on the received signal from the stationary antenna and the transmitted signal. This allows for the determination of the delay difference between the remaining paths and the direct path. Notably, the data received by the mobile antenna are excluded from this process. This observation further demonstrates that maintaining precise relative timing between the stationary and moving antennas is not a requisite. Finally, the spatiotemporal information is combined to achieve the positioning of the collaborative node.

### 2.4. Complete Procedure Analysis

Algorithm 2 presents the complete procedure of this proposed localization technique.
**Algorithm 2:** An Effective Single-Station Cooperative Node Localization Technique Using Multipath Spatiotemporal Information
**Input:** Q data files, each containing K sample points of I/Q data from both moving and stationary antennas at q-th (1≤q≤Q) different angular position; The transmitted waveform of the cooperative target Tx.
**Output:** Estimated coordinates $\left(\hat{x},\hat{y}\right)$ of Tx.1.Execute Algorithm 1 to extract a .mat file containing spatial angles and 
  corresponding power values based on the input data files.2.Load the .mat file to obtain the spatial spectrum and identify the direct path arrival 
  angle ${\hat{\theta }}_{1}$ and L potential values for the reflected path arrival angle ${\hat{\theta }}_{2}$ based 
  on a predefined threshold.3.Calculate the L possible coordinates $\left({\hat{x}}_{l},{\hat{y}}_{l}\right)$ of Tx according to Equation (4).4.Perform matched filtering on the stationary antenna’s received data with the 
  transmitted waveform to obtain the time-domain energy distribution.5.Determine the direct path delay ${\hat{\tau }}_{1}$ and L possible values for the delay differences 
  ${∆\hat{\tau }}_{l}$ between the reflected and main path based on a predefined threshold.6.Match the candidate coordinates $\left({\hat{x}}_{l},{\hat{y}}_{l}\right)$ derived from spatial analysis with the 
  corresponding time delay differences ${∆\hat{\tau }}_{l}$ obtained through temporal analysis 
  based on energy.7.Compute the coordinates $\left(\hat{x},\hat{y}\right)$ of Tx as specified in Equation (14).

Based on both Algorithms 1 and 2, the overall complexity of the proposed positioning scheme can be analyzed from both the time-domain and space-domain perspectives. In the space-domain component, Algorithm 1 processes data from multiple angular positions, with a time complexity of O(Q·K), where Q represents the number of angular positions and K denotes the number of samples at each position. The time complexity of the spatial power spectrum computation is $O(T_{\theta }\cdot Q)$, where the angular range extends from cosθ=−1 to cosθ=1 with a step size of 0.01, resulting in $T_{\theta }=201$ distinct angles. Consequently, the computational burden in the spatial domain is primarily influenced by the number of angular positions Q and the sample size K. The time complexity of the temporal domain primarily arises from performing matched filtering based on cross-correlation between the stationary antenna’s received data and the transmitted signal from target, resulting in a complexity of $O(Q\cdot K^{2})$. In summary, the overall computational complexity of the proposed localization method is $O(Q\cdot K^{2})$. Table 2 presents a comparative analysis of the computational complexity of several existing single-station localization methods.

As illustrated in Table 2, the proposed algorithm exhibits substantially reduced complexity in comparison to other approaches. Indeed, in practical applications, since only a limited number of sampling points and angular positions are typically required, this algorithm’s complexity remains manageable, meeting real-time or near-real-time localization demands.

## 3. Experiments and Discussions

### 3.1. Simulation Experiment

#### 3.1.1. Feasibility Analysis

**Figure 3:**
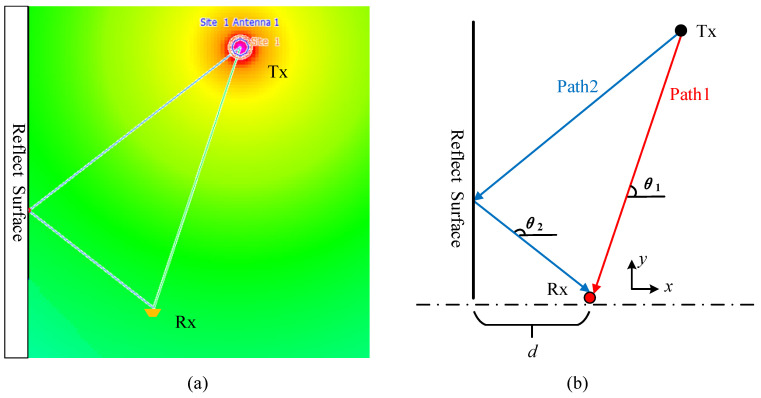
(**a**) Signal propagation diagram simulated by WinProp; (**b**) path propagation analysis for (**a**), where the red path (Path1) represents the direct path, while the blue path (Path2) denotes the reflected path.

First, this paper simulates and verifies the model illustrated in Figure 1 based on the ray propagation model. The signal propagation path is shown in Figure 3, where Figure 3a shows the multipath propagation routes generated by simulating the model using WinProp 2020 software, and Figure 3b provides an analysis diagram of the path propagation situation in Figure 3a. In this setup, a known-position reflective surface is set to simulate the prior geographic environment. Signals from an unknown-position radiation source reach the receiver via paths Path1 and Path2, with spatial azimuth angles $\theta_{1}$ and $\theta_{2}$, respectively.

Simulation conditions: A Cartesian coordinate system is established at the receiver Rx, with the rotation center of the moving antenna as the origin and the horizontal axis to the right as the zero-reference point. The stationary antenna is positioned at $\left(-0.63,\;0.04\right)$, while the target coordinates are set to $\left(0,\;2.95\right)$ (in meters). The distance from Rx to the reflective surface Reflect Surface is d=1.85 m. In the simulation, frequency-modulated continuous wave (FMCW) is selected as the transmitted signal. The baseband signal bandwidth is set to B=100 MHz, with a maximum frequency $f_{1}=110\;MHz$, a minimum frequency $f_{2}=10\;MHz$, a carrier frequency $f_{c}=2.56\;GHz$, a sampling rate $f_{s}=245.76\;Msps$, as well as a number of sampling points SamNum=4096. Assuming that each antenna receives independent, identically distributed Gaussian white noise without amplitude or phase errors, the position of the cooperative radiation source is estimated using the method proposed in this paper.

**Figure 4:**
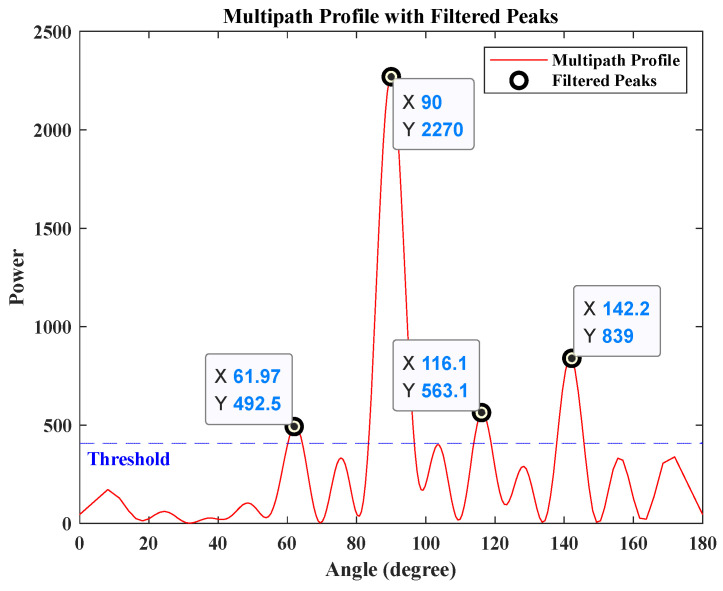
Direction-finding simulation analysis results.

Figure 4 illustrates the multipath profile. Considering the effects of noise and other factors, a threshold is set at one-sixth of the maximum value, and the angles corresponding to the peaks exceeding this threshold are regarded as potential propagation paths of the target in this detection. Given that the direct path has the largest energy, it can be considered that ${\hat{\theta }}_{1}=90{}^{\circ}$, and the possible values of ${\hat{\theta }}_{2}$ are $\left[61.97{}^{\circ},\;116.10{}^{\circ},\;142.19{}^{\circ}\right]$. As illustrated in the signal propagation model in Figure 3, ${\hat{\theta }}_{2}>{\hat{\theta }}_{1}$. According to Equation (4), two possible coordinate values can be derived: $\left(0,\;7.55\right)$ and $\left(0,\;2.87\right)$. To identify the optimal estimate of the target position, a matched filtering operation was performed on the model using the MATLAB R2018b simulation system based on the parameters above.

**Figure 5:**
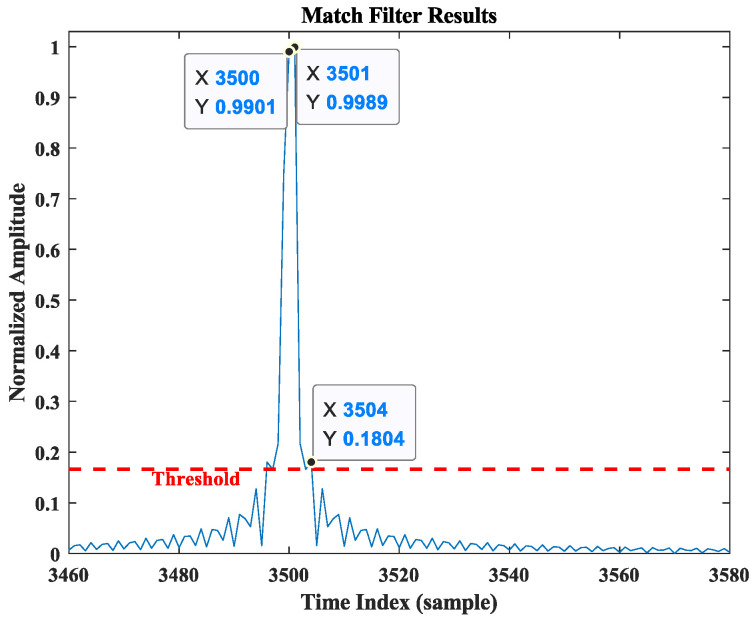
Simulation detection delay spectrum.

In Figure 5, the resulting normalized power distribution over the time index is shown, with a threshold of one-sixth of the maximum value being applied. Due to the direct path having the maximum energy and the minimum delay, only the peak point at the maximum value and the peak points exceeding the threshold on its right are considered. Thus, three target points meeting the criteria can be observed in the figure, with the corresponding relative delay parameters as follows:

(18) $$t_{1}=3500\;samples,\;t_{2}=3501\;samples,\;t_{3}=3504\;samples.$$

Based on the relationship between path energy and time distribution in Figure 5, it can be inferred that $t_{1}$ represents the relative delay of the direct path. Furthermore, the potential time delay difference between the direct path and the reflected path can be calculated as follows:

(19) $${∆\hat{\tau }}_{2,1}=t_{2}-t_{1}=1\;sample,\;{∆\hat{\tau }}_{3,1}=t_{3}-t_{1}=4\;samples.$$

In this simulation, the sampling point interval is $1/f_{s}$, providing a distance resolution of $∆R=c/f_{s}=1.22\;m/sample$ based on the cross-correlation operation. The possible distance differences of the two paths can be calculated as 1.22m and 4.88 m, respectively. According to the analysis in Section 2.2 and the calculations of Equation (14), it can be concluded that the detection value of the cooperative object is $\left(0,\;2.87\right)$, closely matching the initial position set at $\left(0,\;2.95\right)$, with an error of RMSE=0.0785 m.

#### 3.1.2. Influencing Factors Analysis

Conducting a sensitivity analysis on the factors influencing localization accuracy, including antenna placement, environmental changes, and potential sources of error such as multipath interference and noise, proves essential, shaping the overall practicality of the approach and providing key insights for strengthening its robustness in future iterations.

**Figure 6:**
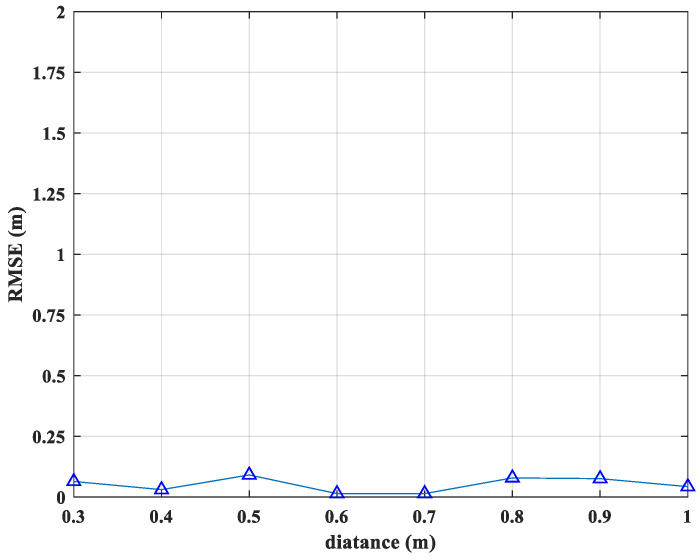
RMSE results versus d.

We first examine the influence of antenna placement on the localization performance with the method proposed in this paper. Given that the Equation (16) has no terms related to the relative position between the two antennas, it can be concluded that the relative positioning of the moving and stationary antennas has no impact on the localization results. A simulation is conducted for further validation, examining the variation in RMSE as the distance d(d>r) between the stationary antenna and the center of rotation of the moving antenna changes. The results, presented in Figure 6, demonstrate that the RMSE remains consistently stable, even with changes in the relative position between the two antennas. As a result, it is pivotal to note that the antenna placement does not affect the final positioning outcome, allowing the antenna positions to be flexibly adjusted in practical applications according to specific requirements.

**Figure 7:**
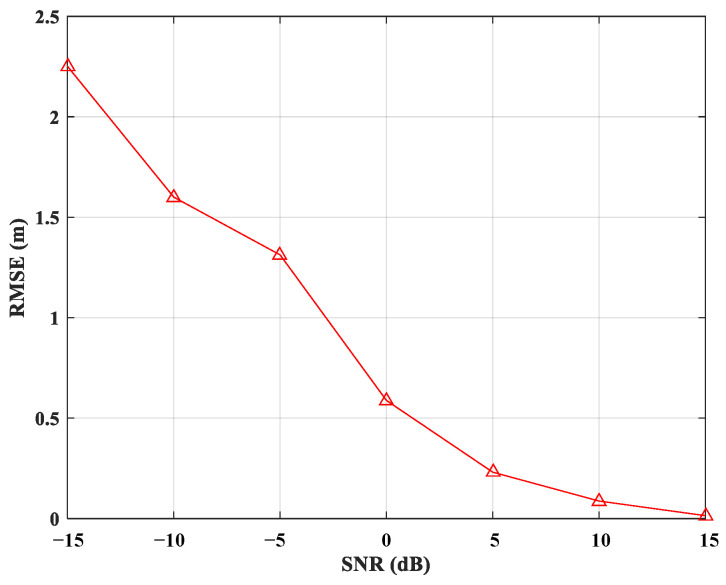
RMSE results versus SNR.

Furthermore, the environmental changes, as well as potential sources of error such as multipath interference and noise, significantly impact the signal-to-noise ratio (SNR) of positioning reception signals, thereby degrading localization performance. We conduct a simulation experiment to observe the variation of RMSE with changes in the SNR, as illustrated in Figure 7. At low SNR, the RMSE is elevated, reflecting reduced localization accuracy. As SNR increases, the error diminishes and gradually converges towards zero. When the SNR exceeds 5 dB, the localization accuracy of our algorithm meets the requirements of most localization applications.

Through simulation analysis, the feasibility of the positioning algorithm proposed in this paper has been demonstrated. To further validate the algorithm’s performance, the following section presents testing in an anechoic chamber, where real data collection and processing are conducted under controlled conditions.

### 3.2. System Design

**Figure 8:**
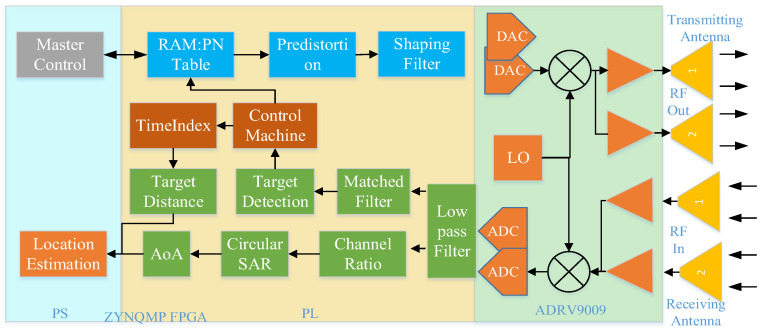
Sensor hardware design overall block diagram.

In order to verify the simulation model and its signal processing algorithm and process in the scenario experiment, this paper developed an RF sensor system, as shown in Figure 8. The system is generally divided into three parts: antenna unit, RF front end, and field-programmable gate array (FPGA) signal processing unit. The antenna unit consists of a 2-transmit and 2-receive configuration with omnidirectional wideband antennas, covering a frequency range from 600 MHz to 6000 MHz.

Collaborative positioning RF sensors are often mounted on outdoor mobile platforms like unmanned vehicles and drones, necessitating compact size and low power consumption. To meet these requirements, this paper designs an RF front-end solution based on the ADRV9009 RF transceiver (Wuhan Qishengtong Technology Co., Ltd., Wuhan, China) integrated chip. The chip supports four independent communication links of 2-receive and 2-transmit channels, enabling the transmission and reception of ultra-wideband RF signals ranging from 75 MHz to 6000 MHz. The core control unit adopts Xilinx’s heterogeneous architecture ZYNQMP FPGA, consisting of resource-rich FPGA programmable logic (PL) and four ARM architecture A53 hard-core processing systems (PSs). Most digital signal processing (DSP) tasks, such as filtering, sequence acquisition and tracking, azimuth angle of arrival calculation, and range estimation, are completed in the core control unit.

**Figure 9:**
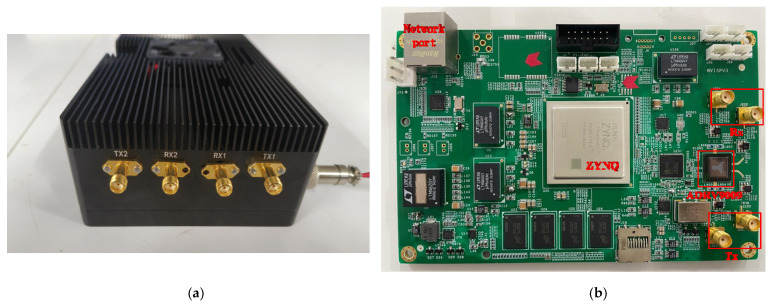
(**a**) Dual-channel RF transceiver; (**b**) physical board of the RF transceiver.

The actual picture of the RF sensor developed in this paper and its internal board diagram are shown in Figure 9a,b, where TX1 and TX2 correspond to two transmitting channels, and RX1 and RX2 represent two receiving channels, respectively. During the experimental validation process, the host computer transmits a known waveform signal through TX1. After being emitted by the transmitting antenna, the signal is received by two antennas at the receiving end and is subsequently sent to the RX1 and RX2 channels of another independent RF sensor for further processing.

### 3.3. Anechoic Chamber Scenario Experiment

**Figure 10:**
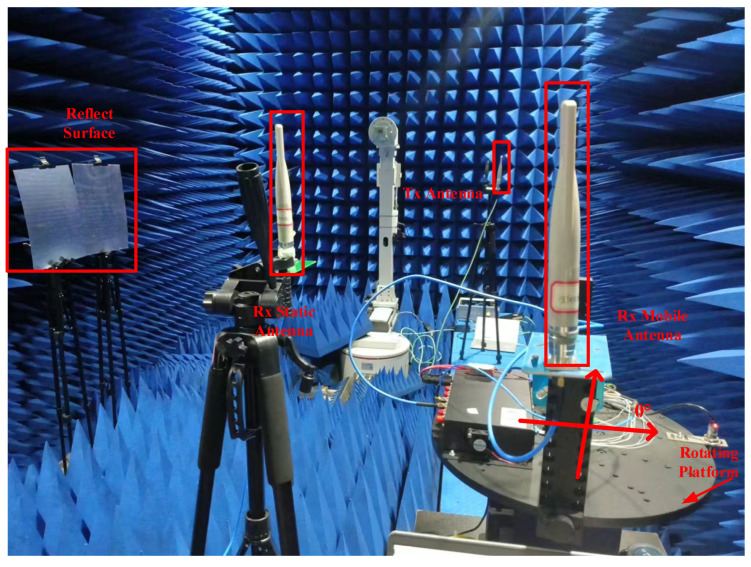
Test equipment and scene diagram.

Subsequently, this paper selects an anechoic chamber environment with weak external multipath interference as the experimental setting, based on the scene layout depicted in Figure 1. As shown in Figure 10, omnidirectional antennas (Wuhan Qishengtong Technology Co., Ltd., Wuhan, China) are used for both transmission and reception, with an antenna gain of 9 dB and a transmission power of 30 dBm. Both the transmitting and receiving devices use dual-channel RF transceivers, operating as described in Section 3.2. The system operates at a frequency of 2.56 GHz. The rotating antenna and RF sensor used for reception are mounted on a device (Wuhan Qishengtong Technology Co., Ltd., Wuhan, China) that allows for seamless continuous high-precision electronic rotation through 360 degrees horizontally. The rotation radius of the antenna is 0.28 m, and the stationary antenna is placed close to the rotating platform. Prior to the commencement of the experiment, it is essential to perform a system calibration. Since this localization framework, involving spatial angle measurement and path delay difference calculation, operates without clock synchronization and the potential impact of time drift is negligible, the system calibration is primarily dedicated to enhancing the precision of angle estimation, with the procedure outlined as follows: First, in the initial setting stage of the system, no reflection paths are set in the scene, and the direct path is mainly considered. At the receiving end, the turntable drives the rotating antenna, recording its angular position every 10 degrees. Data received by both the rotating and stationary antennas is saved at each position, resulting in a total of 36 sets of data. A coordinate system is established, as shown in Figure 10, with the center of the turntable rotation as the origin and the horizontal direction to the right serving as the reference zero point. For a given true incoming wave direction $\alpha_{1}$, the method proposed in Section 2.3 is applied to derive the corresponding angle estimation ${\hat{\alpha }}_{1}$, with the angular error quantified as $\Delta \delta =\left|\alpha_{1}-{\hat{\alpha }}_{1}\right|$. Then, we change the value of $\alpha_{1}$ and repeat the above steps. Ultimately, the average value $\Delta \bar{\delta }$ of the angular discrepancies Δδ is calculated, which is utilized to reflect the intrinsic direction-finding inaccuracies induced by system hardware and other interfering factors, and subsequently employed to correct the operational results, enabling the acquisition of more precise angle estimates, thereby improving the reliability and accuracy of the entire positioning system. During the experiment, the same procedure as during calibration is followed at the receiving end, while at the transmitting end, the target Tx initial position is set to (0, 2.95) m, and the detection target position can be adjusted by moving the position of the transmitting antenna as needed. Another independent RF sensor is used to generate an FMCW signal with a bandwidth of B=100 MHz for transmission. A metal plate is also set up to create a reflection path for obtaining additional information to locate the target, with d=1.85 m.

**Figure 11:**
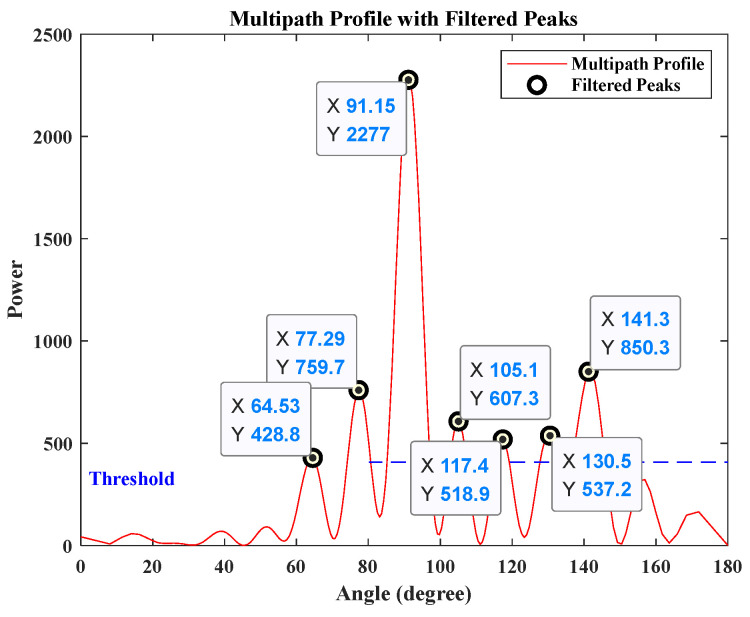
Direction-finding scenario experimental analysis results.

Using MATLAB R2018b, the data received by the antenna is analyzed to create a multipath profile, as shown in Figure 11. In this figure, the horizontal axis represents the spatial angle, while the vertical axis denotes the power spectrum amplitude. Considering the interference caused by reflections and scattering from other metal rods in the experimental scene, one-sixth of the maximum amplitude value is set as the threshold. The horizontal axis values of the peak points exceeding this threshold correspond to the possible AOAs for the detected paths. From this analysis, seven possible values are obtained, as indicated in Figure 11. The angle corresponding to the maximum power value is considered the AOA for the direct path, that is, ${\hat{\theta }}_{1}=91.15{}^{\circ}$. The possible AOAs for the reflected path, ${\hat{\theta }}_{2}$, are $\left[64.53{}^{\circ},\;77.29{}^{\circ},\;105.1{}^{\circ},\;117.4{}^{\circ},\;130.5{}^{\circ},\;141.3^{{}^{\circ}}\right]$. It can be obviously seen from the scene layout in the Figure 10 that ${\hat{\theta }}_{2}>{\hat{\theta }}_{1}$, and the possible values of ${\hat{\theta }}_{2}$ are further filtered to $\left[105.1{}^{\circ},\;117.4{}^{\circ},\;130.5{}^{\circ},\;141.3{}^{\circ}\right]$. Based on Equation (4), four possible target points are obtained, which are then sorted in descending order of energy as follows:

(20) $${pos}_{1}=\left(-0.06,2.92\right){,\hat{\theta }}_{2}=141.3{}^{\circ},\;{pos}_{2}=\left(-0.26,12.79\right),{\hat{\theta }}_{2}=105.1{}^{\circ},\;{pos}_{3}=\left(-0.08,4.23\right){,\hat{\theta }}_{2}=130.5{}^{\circ},\;{pos}_{4}=\left(-0.14,6.88\right){,\hat{\theta }}_{2}=117.4{}^{\circ}.$$

**Figure 12:**
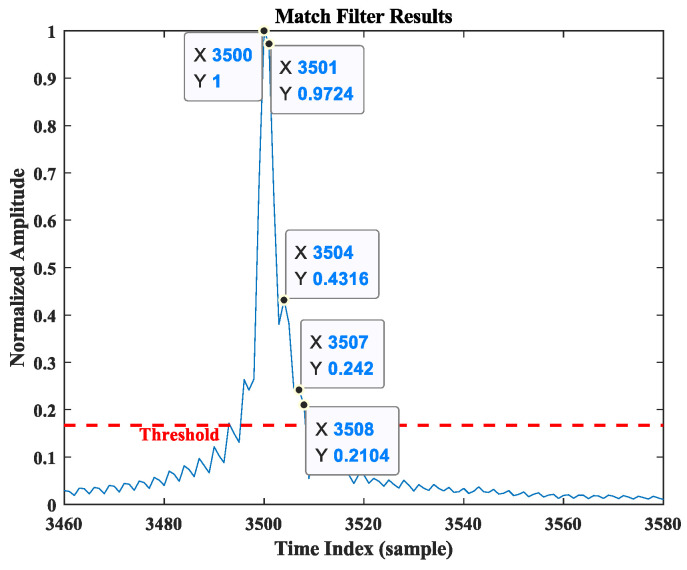
Delay spectrum of scenario experiment.

To identify the optimal solution, cross-correlation is performed between the received data from the stationary antenna and the known data transmitted by the cooperative target. The resulting delay spectrum is shown in Figure 12. In the figure, the horizontal axis represents the time index of the matched filter output sequence, with the unit being the number of sampling points, while the vertical axis denotes the normalized power spectrum amplitude. Since the path energies in both time and spatial domains should have the same relative magnitudes for identical processed data, the information in these domains can be matched based on this. To better observe this phenomenon, we also set the threshold in the delay spectrum at one-sixth of the maximum value. Given that the direct path has the highest energy and shortest delay, the peak point at the maximum value and the peak points to its right that exceed the threshold are selected as the desired observation points. Five relative delay values meeting the criteria are observed in Figure 12. These values are sorted in descending order of amplitude as follows:

(21) $$t_{1}=3500\;samples,\;t_{2}=3501\;samples,\;t_{3}=3504\;samples,\;t_{4}=3507\;samples,\;t_{5}=3508\;samples,$$

where $t_{1}$ is the time index of the direct path. We subtract the time index of $t_{1}$ from the remaining index to obtain the possible delay difference between the two paths in units of sampling point samples:

(22) $${∆\hat{\tau }}_{2,1}=t_{2}-t_{1}=1\;sample,\;{∆\hat{\tau }}_{3,1}=t_{3}-t_{1}=4\;samples,\;{∆\hat{\tau }}_{4,1}=t_{4}-t_{1}=7\;samples,\;{∆\hat{\tau }}_{5,1}=t_{5}-t_{1}=8\;samples.$$

The known distance resolution ∆R is then used to convert the delay difference into seconds. Based on the analysis in Section 2, the possible target positions can be matched with the delay differences to obtain $\left\{\left({pos}_{1},\;{∆\hat{\tau }}_{2,1}\right),\;\left({pos}_{2},\;{∆\hat{\tau }}_{3,1}\right),\;\left({pos}_{3},\;{∆\hat{\tau }}_{4,1}\right),\;\left({pos}_{4},\;{∆\hat{\tau }}_{5,1}\right)\right\}$. According to Equation (14), a numerical solution is performed in MATLAB R2018b, and the detected coordinates of the cooperative object’s position are $\left(-0.058,\;2.922\right)$, which are in close agreement with the actual position parameters of the target $\left(0,\;2.95\right)$, and the measurement error RMSE=0.065 m.

## 4. Conclusions

This paper investigates a single-station passive positioning system for cooperative sensor nodes, utilizing the geometric information of surrounding environmental structures as prior knowledge. By extracting the spatiotemporal information embedded in MPCs, the system constructs multiple virtual observation points for target detection, thereby eliminating the need for large-scale antenna arrays and enabling a simpler, more cost-effective single-station positioning solution with just two antennas. First, the simulation of cooperative nodes within the developed single-station positioning model, driven by multipath spatiotemporal information, is conducted by using the WinProp and MATLAB R2018b tools. The results verify the effectiveness of the algorithm, achieving a positioning accuracy of 0.1 m. Subsequently, the software-defined radio-based positioning system detects the locations of a cooperative transmitting source in an anechoic chamber, maintaining a positioning error within 0.1 m. Notably, this positioning system requires no clock synchronization between the transmitting and receiving devices, nor among the rotating antennas at different angular positions. This design significantly simplifies the system architecture and reduces deployment costs, shedding light on a more practical and versatile approach to single-station positioning for real-world applications.

## Figures and Tables

**Table 1:** Analysis and comparison of existing systems.

Reference	Technique	Research Findings	Challenges
[18]	Maximum likelihood algorithm with multiple transponders assistance	Single-step processing without explicit estimation of TOA/TDOA or AOA	Requirement for multi-antenna array configuration and higher computational complexity
[19]	Fingerprint matching based on hybrid AOA/PDP	Reduced matching complexity, search latency, and storage overhead, with enhanced accuracy	Requires complex matrix operations and multi-antenna access points
[20]	Exploiting near-field effects of XL arrays	Achieve superior accuracy and robustness with similar complexity compared with benchmark methods	Similarly demand advanced matrix decomposition and large-scale antenna arrays
[21]	Hybrid AOA/TOA method leveraging 5G signals	Improved positioning accuracy	Require time and frequency synchronization, along with spatial spectrum computation and multi-antenna configuration.
Proposed work in this paper	AOA/TDOA hybrid method based on multipath	Require only two antennas, with no complex calculations or time/frequency synchronization, while maintaining high accuracy	Future work will optimize this proposed approach for high-accuracy localization in complex, NLOS environments

**Table 2:** Comparison of computational complexity across methods.

Reference	Computational Complexity	Notes
[18]	$O(L^{3})$	L: number of passive transponders
[19]	$O((\Gamma N+N_{g})M^{2})$	Γ: number of OFDM symbols N: number of subcarriers M: number of antennas $N_{g}$: number of grids searched
[20]	$O((2L-1)N_{t}N_{g}N_{Ref})$	L: maximum delay compensation $N_{t}$: number of antennas $N_{g}$: length of the cyclic prefix (in sampling points) $N_{Ref}$: total number of reference points
Proposed method in this paper	$O(Q\cdot K^{2})$	Q: number of angular positions K: number of samples at each position

## Data Availability

All of the required data are available in the manuscript. We do not have any data to share.
